# Ethnomedicinal plants used for treatment of snakebites in Tanzania – a systematic review

**DOI:** 10.1080/13880209.2022.2123942

**Published:** 2022-10-07

**Authors:** Neema Gideon Mogha, Olivia John Kalokora, Halima Mvungi Amir, David Sylvester Kacholi

**Affiliations:** Department of Biological Sciences, Dar es Salaam University College of Education, University of Dar es Salaam, Dar es Salaam, Tanzania

**Keywords:** Antivenin, envenomation, ethnobotany, herbal remedies, traditional medicine, medicinal plants

## Abstract

**Context:**

Snake envenomation is one of the neglected health problems in Tanzania. Since most people, especially in rural areas, suffer from its burden, their cases are not documented due to reliance on medicinal plants. Despite the pivotal role of medicinal plants in treating snakebites, there is a paucity of information.

**Objective:**

This review documents medicinal plants used to treat snakebites in Tanzania.

**Materials and methods:**

A systematic search using electronic databases such as PubMed, Google Scholar, Scopus, Science Direct and grey literature was conducted to retrieve relevant information on medicinal plants used to treat snakebites in Tanzania. The review was conducted as per the Preferred Reporting Items for Systematic Reviews and Meta-Analyses (PRISMA) statement. The obtained information from 19 published articles was organized and analysed based on citation frequency.

**Results:**

A total of 109 plant species belonging to 49 families are used as snakebite antivenom in Tanzania. Fabaceae had the highest number of medicinal plants (19.3%). The dominant plant growth forms were trees (35%) and shrubs (33%). Roots were the most frequently used plant part (54%), followed by leaves (26%) and bark (11%). *Annona senegalensis* Pers. (Annonaceae), *Dichrostachys cinerea* (L.) (Fabaceae), *Suregada zanzibariensis* Baill. (Euphorbiaceae), *Antidesma venosum* E.Mey. ex Tul. (Phyllanthaceae), *Cissampelos pareira* L. (Menispermaceae) and *Dalbergia melanoxylon* Guill. & Perr. (Fabaceae) were the most cited medicinal plants.

**Conclusions:**

Tanzania has diverse plants used for snakebite treatment; a few have been analysed for their bioactive components. Further study of the phytochemicals may provide scientific information to develop snakebite drugs.

## Introduction

Globally, snake envenomation is considered a neglected disease and a significant public health concern (World Health Organization (WHO) 2007). About 5.5 million people are envenomed annually, whereas 9% of the cases are reported in Africa (Chippaux 1998; Giovannini and Howes 2017). Of the reported cases globally, 36% die due to snakebites, and about 7% survive permanent injuries (WHO 2007; Omara 2020). These figures can be lower than the truth because most snakebite incidences occur in rural areas where there are insufficient health facilities, and most cases are not recorded (Zolfagharian and Dounighi 2015; Yirgu and Chippaux 2019; Omara 2020). In sub-Saharan Africa, about one million cases of snake envenomation are reported annually, causing 2% of death cases and 1% get permanent injuries (Chippaux 2015). In East Africa, 108 and 151 cases of snakebite were reported in Uganda (Wangoda et al. 2004) and Kenya (Snow et al. 1994), respectively. In Tanzania, there are no proper records on snakebite cases despite diverse types of snakes (Chippaux 2011; Kipanyula and Kimaro 2015).

Venomous snakes are found in most parts of the world, in all climatic conditions except in frozen environments and at higher altitudes (WHO 2007; Kasturiratne et al. 2008). Africa alone is a home of 400 different snake types, whereby nearly 50% are found in East Africa. Some of them are black mamba (*Dendroaspis polylepis* Günther (Elapidae)), spitting cobra (*Naja nigricollis* Hallowell (Elapidae)), Rufous-beaked snake (*Ramphiophis rostratus* Peters (Psammophiidae)), puff adder (*Bitis arietans* Parker (Viperidae)) and green mamba (*Dendroaspis jamesoni* Traill (Elapidae)) (Kipanyula and Kimaro 2015; Omara 2020). Snakebites are life-threatening due to the scarcity of proven medication. Although antivenom serum has been developed as a lifesaving option, it is associated with the development of immediate or delayed hypersensitivity (anaphylaxis or serum sickness) and does not avert local tissue damage (Maya Devi et al. 2002). For example, an antidote such as immunoglobulin G produced in horses could react to serum and cause sickness, renal failure and anaphylaxis (Cannon et al. 2008; Giovannini and Howes 2017). Still, antivenom administration is considered chiefly a definitive treatment for snakebites. Other treatments include respiratory support therapy, surgical of affected necrosis tissues or even amputation (Cannon et al. 2008; de Moura et al. 2015).

Regardless of the funding issues, there is a paucity of snake venom antiserum in most African countries, predominantly rural areas. Tanzania faces a similar problem that makes the rural inhabitants depend on traditional medicines, particularly herbal remedies (Maregesi et al. 2013). Other reasons for reliance on traditional medication are distance to medical facilities, poor infrastructure, storage conditions, scarcity of antidotes in hospitals, restricted application, traditional beliefs, and the high cost of antivenom and modern facilities (Giovannini and Howes 2017; Steinhorst et al. 2021; Kacholi and Amir 2022). Therefore, the use of medicinal plants in addressing snakebite problems has been increasing in the modern era due to their safety, effectiveness, cultural preferences, inexpensiveness, abundance and availability (Maregesi et al. 2013; Omara et al. 2020). Despite the critical role of medicinal plants in combating snakebite problems, there is no specific ethnobotanical study that has compiled data on medicinal plants used to manage snakebites in Tanzania. Thus, this review fills that gap by documenting medicinal plants used in various parts of the country to treat snakebites.

## Methods

### Description of the country

Tanzania is a country located in East Africa, covering an area of 947,303 km^2^. It is bordered in the North by Uganda, South by Malawi and Mozambique, Northeast by Kenya, East by the Indian Ocean and Comoro Island, Southwest by Zambia, and West by Burundi, Rwanda and the Democratic Republic of Congo. The country is estimated to have about 56.31 million population, making it the second-most-populous country south of the equator after South Africa. The population comprises over 120 ethnic groups with different beliefs and cultural practices. The great African lakes are partly within this country. Lake Victoria, Africa’s largest lake, is located to the north; Lake Tanganyika, Africa’s deepest lake, is to the west, and Lake Nyasa lies to the south. Africa’s highest mountain, Mount Kilimanjaro, is found on the north-eastern side of the country.

### Literature search strategy

**Figure 1.**
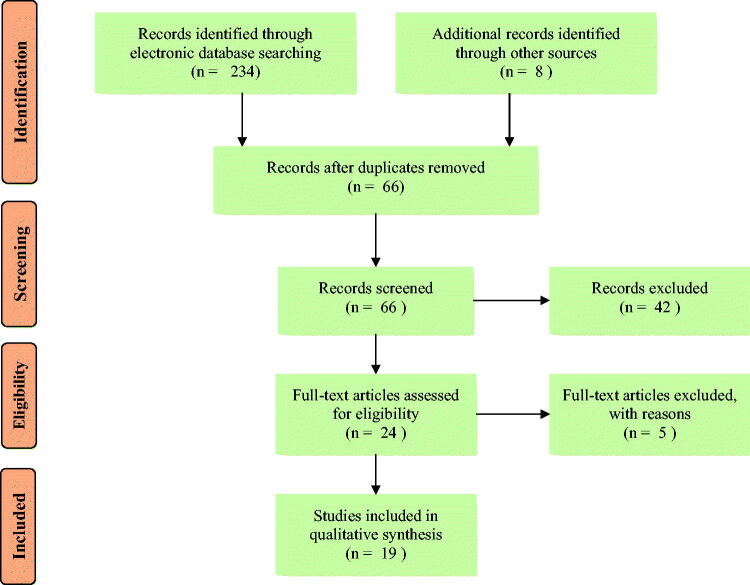
PRISMA study flow diagram for the ethnomedicinal data against snakebites.

This systematic review has compiled information on ethnomedicinal plants used to treat snakebites in different parts of Tanzania. The study was conducted following the recommendations stated in the Preferred Reporting Items for Systematic Reviews and Meta-Analyses (PRISMA) statement (Liberati et al. 2009). The PRISMA flow diagram is presented in Figure 1. A web-based literature search was carried out using various electronic databases, including Google Scholar, Web of Science, African Journals Online (AJOL), Science Direct, Scopus, PubMed, Wiley online library and grey literature to access relevant studies. The following search terms and combinations were used to gather relevant studies; medicinal plants, traditional medicines, ethnomedicine, ethnopharmacology, ethnobotany, alternative medicine, antivenin plants, antivenom, antitoxin, antiophidian, snake antidotes, antisera, snakebite, snake envenomation and Tanzania.

All the searches were conducted independently in all the databases, and only articles published and theses or dissertations having any of the above key terms were considered. The studies written in the English language were only searched and considered. Finally, Tanzanian traditional medicinal plants exclusively utilized to treat snakebites were selected. Records from outside Tanzania, ethnoveterinary studies, pharmacological studies and reviewed articles were excluded from the present study. Also, the studies with no scientific names and the plant parts used were excluded. Studies that possessed required information, such as family name, scientific name, local name, growth habits, method of preparation (if available) and route of administration (if available), were extracted. The precision of the botanical names was also searched and confirmed in botanical databases such as International Plant Names Index (https://www.ipni.org) and Tropicos (https://www.tropicos.org).

### Data analysis

Microsoft Excel software (Redmond, WA) was employed to analyse the frequency distribution of families, plant parts, growth forms and routes of administration. Moreover, the distribution in regions where the medicinal plants were reported was analysed. The results are presented in figures and tables.

## Results and discussion

### Distribution of medicinal plants

**Figure 2.**
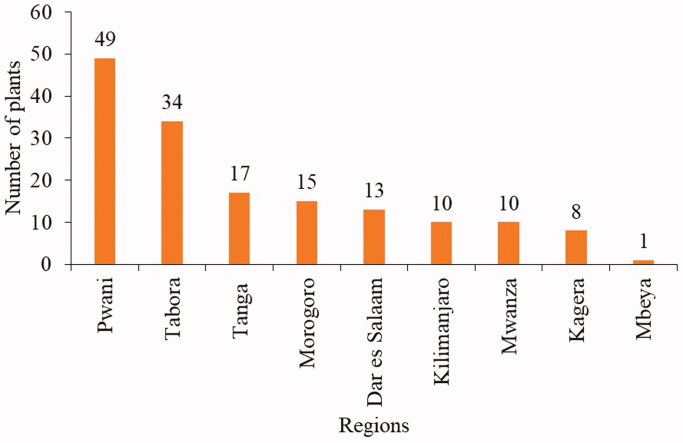
Distribution of medicinal plants across nine Tanzanian regions.

This study has used 19 ethnobotanical studies from nine regions in the country. This indicates that studies on the prevalence of snakebites in different regions of Tanzania are limited (Kipanyula and Kimaro 2015). The regions with the highest ethnomedicinal records were Pwani (49 species) and Tabora (34 species). The remaining regions had less than 17 medicinal plants (Figure 2). The high number of reported antivenin plants in the Pwani and Tabora regions indicates that the locals in the two regions have good indigenous knowledge of the use of plants, and perhaps the regions have many snakebite incidences.

### Diversity of medicinal plants

This review reports 109 plant species belonging to 49 families to manage snakebite problems (Table 1). Globally, the highest number of medicinal plants used against snakebites was reported from India (Upasani et al. 2017), while in sub-Saharan Africa, Ethiopia (Yirgu and Chippaux 2019) recorded the highest number of plants, followed by Uganda (Omara et al. 2020) and Kenya (Omara 2020). This is probably because the highest number of ethnobotanical studies and reviews have been conducted in these countries, making the data more accessible. The majority of the documented plants in this review belong to the family Fabaceae 21 (19.3%), followed by Euphorbiaceae nine (8.3%), Rubiaceae seven (6.4%), Asteraceae and Combretaceae, each with four (3.7% each). The remaining 43 families were represented by less than four species each. The most cited plant species were *Annona senegalensis* Pers. (Annonaceae), *Dichrostachys cinerea* (L.) (Fabaceae), *Antidesma venosum* E.Mey. ex Tul. (Phylanthaceae), *Cissampelos pareira* L. (Menispermaceae) and *Dalbergia melanoxylon* Guill. & Perr. (Fabaceae) suggesting considerable potential for possessing snake envenomation bioactive compounds that can be isolated and combined with commercial antiserum to prepare snakebite antidotes. Details on all medicinal plant species with their respective family, local names, growth form, part used and the region in which they were reported are summarized in Table 1.

**Table 1.** List of medicinal plants used for treatment of snakebites in rural communities of Tanzania.

Family	Species name	Local name	Growth form	Parts used	MoP and RoA	Reference
Acanthaceae	*Crassocephalum mannii* (Hook. f.)	Mgangogango	Shrub	Leaves	Decoction drunk	Maregesi et al. (2013)
	*Justicia heterocarpa* L.	Mwidu	Herb	Roots, leaves	Pounded parts taken orally	Amri and Kisangau (2012)
	*Thunbergia alata* Bojer ex Sims	Nyakatao	Herb	Leaves	Powdered leaves mixed with water to give a paste which is then applied in a small incision made on the bite	Maregesi et al. (2013)
Amaranthaceae	*Aerva lanata* (L.) Juss. ex Schult.	Kambunyenye	Herb	Roots	Not specified	Chhabra et al. (1987)
Anacardiaceae	*Lannea schimperi* (Hochst. ex A.Rich) Engl.	Mgumbu	Tree	Roots	Crushed and applied to the bite	Augustino et al. (2011)
	*Ozoroa mucronata* (Krauss) R. & A. Fern.	Mgombokilangu	Tree	Leaves	Not specified	Chhabra et al. (1987)
Annonaceae	*Annona senegalensis* Pers.	Mtopetope	Tree	Roots, bark, leaves	Decoction drunk	Augustino et al. (2011), Kacholi (2014, 2020), Maregesi et al. (2013), Ruffo (1991)
	*Friesodielsia obovata* (Benth.) Verdc.	Msalasi	Shrub	Roots	Crush and then massage the affected area	Augustino et al. (2011), Ruffo (1991)
	*Uvaria acuminata* Oliv.	Msofu	Shrub	Roots	Decoction drunk	Chhabra et al. (1987)
Apocinaceae	*Xylopia longipetala* De Wild & T. Durand	Mlavilira	Tree	Bark	Not specified	Kacholi (2014)
Araceae	*Gonatopus boivinii* (Decne.) Engl.	Mzulu	Herb	Roots	Powdered roots applied to the bite	Chhabra et al. (1987)
Aristolochiaceae	*Aristolochia spp.*	Kilikamo	Climber	Roots	Not specified	Ruffo (1991)
Asteraceae	*Blephurispermum zanguebaricum* Oliv. & Hiern	Msekele	Shrub	Roots	Decoction drunk	Chhabra et al. (1989)
	*Conyza canadensis* L. Cronq.	Akamwisanga	Herb	Whole plant	Decoction drunk	Maregesi et al. (2013)
	*Spilanthes mauritiana* (Rich. ex Pers.) DC	Mtango	Herb	Whole plant	Not specified	Chhabra et al. (1989)
	*Vernonia amygdalina* Del.	Mtugutu	Shrub	Leaves	Leaves chewed, and extract swallowed	Chhabra et al. (1989)
Bignoniaceae	*Markhamia obtusifolia* (Baker) Sprague	Ng’ubu	Tree	Roots	Chew and swallow the extracts	Chhabra and Mahunnah (1994)
Burseraceae	*Commiphora africana* (A.Rich.) Engl.	Mutonto	Tree	Bark	Crush and massage the affected area	Augustino et al. (2011)
Cannoraceae	*Byrsocarps orientalis* (Baill.) Baker	Mpandaradu	Herb	Roots	Dried roots are burnt, mixed with powdered charcoal and tobacco, and the infusion drunk	Chhabra et al. (1989)
Capparaceae	*Capparis tomentosa* Lam.	Mtungulang’osa	Tree	Leaves	Not specified	Chhabra et al. (1989)
	*Thylachium africanum* Lour.	Mtomoni	Tree	Roots	Not specified	Chhabra et al. (1989)
Celastraceae	*Maytenus senegalensis* (Lam.) Exell	Mwambangoma	Tree	Roots	Not specified	Ruffo (1991), Chhabra et al. (1989)
Combretaceae	*Combretum apiculatum* Sond.	Muhuluka	Tree	Roots	Not specified	Chhabra et al. (1989)
	*Combretum collinum* Fresen.	Mulandala	Tree	Roots	Not specified	Ruffo (1991)
	*Combretum obovatum* F. Hoffm	Vugoveko	Tree	Roots	Crush roots, then massage the affected part	Augustino et al. (2011)
	*Combretum zeyheri* Sond.	Musana	Tree	Leaves	Crush roots, then massage the affected part	Augustino et al. (2011)
Convolvulaceae	*Jacquemontia paniculata* (Burm. f.) Hall. F.	Mwidilimbwi	Herb	Leaves	Fresh pounded leaves are applied to the bite	Chhabra et al. (1989)
Cucurbitaceae	*Momordica foetida* Schumach	Ruhunduhundu	Herb	Leaves	Not specified	Chhabra et al. (1989)
Dilleniaceae	*Tetracera boiviana* Baill.	Mpinga	Tree	Roots, leaves	Powdered roots mixed with water and taken orally, while leaves are crushed and juice drunk	Chhabra et al. (1989)
Ebenaceae	*Diospyros fischeri* Guerke	Mufubata	Shrub	Roots	Crushing, then massage the affected part	Augustino et al. (2011)
	*Diospyros usambarensis* F. White	Mwiloiloi	Shrub	Roots	Not specified	Chhabra et al. (1989)
	*Euclea divinorum* Hiern.	Mdaa	Shrub	Leaves	Crush leaves and massage the affected part	Augustino et al. (2011)
Euphorbiaceae	*Acalypha fruticosa* Forssk.	Mfulwe	Shrub	Aerial parts	Crushed and applied on incisions made on the bitten area	Chhabra et al. (1990b), Hedberg et al. (1983a)
	*Cyathogyne bussei* Pax	Mzidishanguvu	Shrub	Roots	Not specified	Chhabra et al. (1990b)
	*Euphorbia candelabrum* Tremaux	Ganga	Tree	Roots	Not specified	Hedberg et al. (1983a)
	*Euphorbia grantii* Oliv.	Mudulansongo	Tree	Roots	Crushing, then massage the affected part	Augustino et al. (2011)
	*Euphorbia hirta* L.	Mziwaziwa	Herb	Roots	Crushing, then massage the affected part. Decoction drunk	Augustino et al. (2011), Chhabra et al. (1990b)
	*Euphorbia tirucalli* L.	Mnyaa	Shrub	Roots	Crushing, then massage the affected part	Augustino et al. (2011), Ramathal and Ngassapa (2001)
	*Phyllanthus reticulatus* Poir.	Mkwambamazi	Shrub	Roots	Infusion drunk	Chhabra et al. (1990b)
	*Securinega virosa* (Roxb. ex Wind.) Pax & K. Hoffm.	Msokote	Shrub	Roots, fruits	Infusion drunk	Hedberg et al. (1983a)
	*Suregada zanzibariensis* Baill.	Mdimumwitu	Shrub	Roots, leaves	Pounded, then massage the affected part after a small incision is made and decoction drunk	Augustino et al. (2011), Augustino and Gillah (2005), Chhabra et al. (1990b), Hedberg et al. (1983a)
Fabaceae	*Abrus precatorius* L.	Lufambo	Herb	Roots	Chewed and extracts swallowed	Chhabra et al. (1990a), Hedberg et al. (1983b)
	*Acacia brevispica* Harms	Msewa	Tree	Roots	Decoction drunk	Chhabra et al. (1990a)
	*Acacia polyacantha* Willd. subsp. campylacantha (Hochst. ex A. Rich.) Brenan	Mgunga	Tree	Roots	Infusion drunk, and the same is applied to the bite	Chhabra et al. (1990a), Hedberg et al. (1983a)
	*Afzelia quanzensis* Welw.	Mkongo	Tree	Roots	Not specified	Chhabra et al. (1987)
	*Brachystegia boehmii* Benth.	Muyombo	Tree	Leaves	Pound and massage the affected part. Decoction is drunk	Augustino et al. (2011)
	*Brachystegia spiciformis* Benth.	Muguluka	Tree	Bark	Pound and massage the affected part. Decoction is drunk	Augustino et al. (2011)
	*Pterocarpus angolensis*	Mninga	Tree	Roots	Chewed and applied on the bite	Augustino et al. (2011)
	*Cassia abbreviata* Oliv.	Muzoka	Shrub	Roots	Decoction drunk	Chhabra et al. (1987)
	*Cassia alata* L.	Mchingu	Shrub	Leaves	Not specified	Chhabra et al. (1987)
	*Cassia occidentalis* L.	Mlingajini	Herb	Leaves	Decoction drunk	Chhabra et al. (1987)
	*Dalbergia melanoxylon* Guill. & Perr.	Mpingo	Tree	Bark, leaves	Fresh leaves are pounded, and juice is drunk	Chhabra et al. (1990a), Ruffo (1991), Salinitro et al. (2017)
	*Dichrostachys cinerea* (L.) Wight et Arn. subsp. africana Brenan et Brummitt	Mkulagembe	Shrub	Roots, bark, leaves	Leaves are chewed, and the paste is applied to the bite. Roots and bark are chewed or macerated and put on the bite	Chhabra et al. (1990a), Hedberg et al. (1983a), Kacholi (2020), Ruffo (1991)
	*Erythrina abyssinica* DC.	Mukalalwanhuba	Tree	Roots	Crushing and then massaging the affected part and sap is used as an antidote	Augustino et al. (2011)
	*Indigofera arrecta* Hochst. A. Rich.	Umusororo	Herb	Whole plant	The plant is dried and ground into a powder, then applied to the affected part	Ramathal and Ngassapa (2001)
	*Isoberlinia angolensis* (Welw. ex Benth.) Hoyle & Brenan	Muva	Shrub	Bark	Chewed and pasted on the bite	Augustino et al. (2011), Ruffo (1991)
	*Lonchocarpus capassa* Rolfe	Mfumbili	Tree	Whole plant	Not specified	Chhabra et al. (1990a)
	*Millettia usaramensis* Taub.	Mhafa	Tree	Roots	Decoction drunk	Chhabra et al. (1990a)
	*Oormocarpum trachycarpum* (Taub.) Harms	Mukondwanhuli	Shrub	Leaves	Crushed, then applied to the bite	Augustino et al. (2011)
	*Pericopsis angolensis* (Baker) Meeuwen	Muvunga	Tree	Leaves	Not specified	Ruffo (1991)
	*Phaseolus radiatus* L.	Ebhisanda	Climber	Seeds	Make powder, then mix with honey and rub to the affected area after a small razor incision	Maregesi et al. (2013)
	*Piliostigma thonningii* (Schum.) Milne-Redh.	Mutindambogo	Tree	Bark	Crushing, then massage the affected part	Augustino et al. (2011)
Flacourtiaceae	*Flacourtia indica* (Burm.f.) Merr.	Mupugusa	Tree	Roots	Crushing, then massage the affected part	Augustino et al. (2011)
Gramineae	*Brachiaria reptans* (L.) Gardner et C.E. Hubbard	Lukoka	Grass	Whole plant	The whole dried plant is burned and the ashes applied to the bite	Chhabra et al. (1990b)
	*Pennisetum purpureum* Schum.	Urubingo	Herb	Whole plant	The plant is dried and ground into a powder, then applied to the affected part	Ramathal and Ngassapa (2001)
Icacinaceae	*Hoslundia opposita* Vahl	Mvulavula	Shrub	Leaves	Crushed and applied to the bite	Chhabra et al. (1990b), Hedberg et al. (1983a)
	*Ocimum basilicum* Linn.	Mvumbasi	Herb	Roots	Not specified	Chhabra et al. (1990b)
Lamiaceae	*Leonotis mollissima* Guerke	Kitalelante	Herb	Leaves	Not specified	Chhabra and Mahunnah (1994), Hedberg et al. (1983a)
	*Rotheca myricoides* (Hochst.) Steane & Mabb.	Umukuzanyana	Herb	Bark	Pounded, then massage the affected part	(Ramathal and Ngassapa (2001)
Lauraceae	*Cassytha filiformis* Linn.	Mlangamia	Herb	Whole plant	Not specified	Chhabra et al. (1990b)
Loganiaceae	*Strychnos spinosa* Lam.	Mtonga	Shrub	Roots	Pounding, then massage the affected part or chew and swallow	Augustino et al. (2011), Chhabra et al. (1990b), Hedberg et al. (1983a)
Malvaceae	*Hibiscus micranthus* L. f.	Muambe Msase	Shrub	Roots	Not specified	Chhabra et al. (1990b), Hedberg et al. (1983a)
	*Sida rhombifolia* L.	Rushuhya	Herb	Roots	Decoction drunk	Chhabra et al. (1990b), Hedberg et al. (1983a)
	*Waltheria indica* L.	Ikumbo-lyaza	Shrub	Roots	Not specified	Ruffo (1991)
Menispermaceae	*Cissampelos mucronata* A. Rich.	Msangwi	Climber	Roots	Root scrapings are taken orally and also rubbed into scars at the place of the bite	Chhabra et al. (1990b)
	*Cissampelos pareira* L. var. orbiculata (DC.) Miq.	Mukuluwanti	Climber	Roots	Infusion drunk	Chhabra et al. (1990b), Hedberg et al. (1983a), Ruffo (1991)
Moraceae	*Ficus natalensis* Hochst.	Mlumba	Tree	Roots	Not specified	Chhabra et al. (1990a)
Ochnaceae	*Ochna schweinfurthiana* F. Hoffm.	Kavulwampako	Shrub	Roots	Not specified	(Ruffo (1991)
Olacaceae	*Ximenia caffra* Sond.	Mnembwa	Tree	Roots	A decoction is taken orally	Augustino et al. (2011), Maregesi et al. (2013)
Oleaceae	*Jasminum fluminense* Vell.	Muhafu	Climber	Roots, Leaves	Pounded and compressed in cotton cloth to get the juice which is taken orally	Chhabra et al. (1990a), Maregesi et al. (2013)
	*Schrebera trichoclada* Welw.	Muputika	Shrub	Bark	Pound and massage the affected part	Augustino et al. (2011)
Orchidaceae	*Ansellia africana* Lindl.	Inyazya	Herb	Bark	Not specified	Ruffo (1991)
Passifloraceae	*Adenia gummifera* (Harv.) Harms	Zokambago	Climber	Roots	Chewed and extracts swallowed	Hedberg et al. (1983b)
Phylanthaceae	*Antidesma venosum* E. Mey. ex Tul.	Musekela, Mnyembelezu, Enjanemeno	Shrub	Roots	Pound and massage the affected part	Augustino et al. (2011), Chhabra et al. (1993), Hilonga et al. (2019), Ruffo (1991)
Poaceae	*Sporobolus pyramidalis* Beauv.	Chinswi	Grass	Roots	Decoction taken orally thrice a day	Maregesi et al. (2013)
Polygalaceae	*Securidaca longepedunculata* Fresen	Msigi	Shrub	Roots, bark, leaves	Decoction of roots, leaves and stem bark drunk	Chhabra et al. (1991), Hedberg et al. (1983b)
Ranunculaceae	*Clematis brachiata* Thunb.	Tambariko	Climber	Leaves	Not specified	Chhabra et al. (1991)
Rhamnaceae	*Ziziphus mucronata* Wild.	Kagovole	Tree	Roots	Infusion drunk	Hedberg et al. (1983b), Ruffo (1991)
Rubiaceae	*Agathisanthemum bojeri* Klotzsch	Mwima	Herb	Leaves	Not specified	Chhabra et al. (1991)
	*Catunaregam nilotica* (Stapf) Tirvengadum	Mtutuma	Shrub	Roots	Infusion drunk	Chhabra et al. (1991), Hedberg et al. (1983b)
	*Catunaregam spinosa* (Thunb.) Tirvengadum	Mwachanguku	Shrub	Roots	Not specified	Chhabra et al. (1991)
	*Gardenia ternifolia* Schumach. & Thonn. subsp. *jovistonantis* (Welw.) Verde.	Mvulang’ondo	Shrub	Roots	Not specified	Chhabra et al. (1991)
	*Hymenodictyon parvifolium* Oliv.	Muginya	Tree	Roots	Infusion drunk	Chhabra et al. (1991)
	*Rothmannia engleriana* (K. Schum.) Keay	Mkondokondo	Tree	Roots	Not specified	Ruffo (1991)
	*Vangueria infausta* Burch. ssp. *infausta*	Msada	Shrub	Roots	Not specified	Chhabra et al. (1991), Kacholi (2020)
Rutaceae	*Citrus limon* (L.) Osbeck	Mlimao	Tree	Leaves	Not specified	Augustino and Gillah (2005), Ruffo (1991)
	*Toddalia asiatica* (L.) Lam.	Mkananga	Shrub	Roots	Infusion drunk	Chhabra et al. (1991)
	*Toddaliopsis sansibarensis* (Engl.) Engl.	Mndizi	Shrub	Roots	Not specified	Chhabra et al. (1991)
Sapindaceae	*Paullinia pinnata* L.	Lugoto	Climber	Leaves	Not specified	Chhabra et al. (1991)
Solanaceae	*Solanum incanum* L.	Nyanyapori	Herb	Roots, fruits, leaves	Dried and pounded and then applied to the bite. Sap and juice from fruits are applied directly to the bite.	Ramathal and Ngassapa (2001), Chhabra et al. (1993), Hedberg et al. (1983b)
Sterculiaceae	*Sterculia africana* (Lour.) Flori	Muhozya	Tree	Bark	Not specified	Ruffo (1991)
	*Sterculia appendiculata* K. Schum	Mgude	Tree	Bark	Decoction drunk	Chhabra et al. (1993), Hedberg et al. (1983b)
Thymelaeaceae	*Synaptolepis kirkii* Oliv.	Mjanungu	Climber	Roots	Not specified	Chhabra et al. (1993)
Tiliaceae	*Grewia bicolor* Juss	Mkele/Mkole Mkoma	Shrub	Roots	Infusion drunk	Chhabra et al. (1993), Hilonga et al. (2019)
	*Grewia fallax* K. Schum	Mkarati	Shrub	Roots	Crushed roots and wipe on the bite	Chhabra et al. (1993)
	*Triumfetta rhomboidea* Jacq.	Mfungang’ombe	Herb	Leaves	Powdered leaves are tied to the affected site and decoction drunk once a day	Chhabra et al. (1993), Maregesi et al. (2013)
Umbeliferae	*Steganotaenia araliacea* Hochst.	Msumi	Tree	Roots	Pound and then massage the affected part	Augustino et al. (2011), Chhabra et al. (1993), Hedberg et al. (1983b)
Verbenaceae	*Premna chrysoclada* (Bojer) Gurke	Mtulanvha	Shrub	Roots, leaves	Not specified	Chhabra et al. (1993), Hedberg et al. (1983b)
Vitaceae	*Cissus hildebrandtii* Gilg.	Mtuha	Herb	Leaves	Not specified	Chhabra et al. (1993)

MoP: mode of preparations; RoA: route of administration.

The predominance of the family Fabaceae was similarly reported in Uganda (Omara et al. 2020), Ethiopia (Yirgu and Chippaux 2019) and India (Upasani et al. 2017). This family’s highest usage is associated with richness in terms of species and comprehensive coverage of ecological habitats (Kadir et al. 2015; Ajao et al. 2019). The family Fabaceae is characterized by active phytochemical compounds such as tannins, phenols and alkaloids (Luís et al. 2011; Żarnowski et al. 2014). Other families reported in this study were also reported to possess antivenin potential for treating or avoiding snakebites in other countries within and outside Africa. For example, Aristolochiaceae and Lamiaceae in Djibouti (Hasan et al. 2016; Yirgu and Chippaux 2019), Acanthaceae, Apocynaceae, Asteraceae, Euphorbiaceae, Moraceae, Rubiaceae and Rutaceae in India (Upasani et al. 2017), Bangladesh (Hasan et al. 2016) and Central America (Giovannini and Howes 2017), Euphorbiaceae, Asteraceae, Amaryllidaceae and Solanaceae in Uganda (Omara et al. 2020), and Malvaceae, Annonaceae, Combretaceae and Lamiaceae in Kenya (Omara 2020).

### Growth forms of medicinal plants

**Figure 3.**
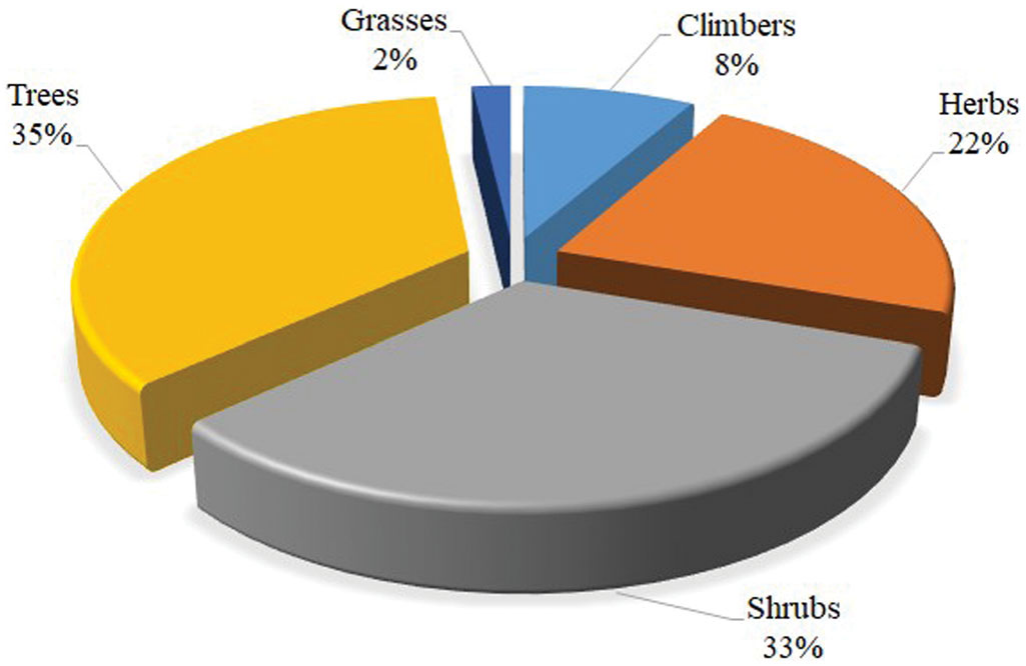
Growth forms of plants used for the treatment of snakebites.

Among the reported medicinal plants in this review, trees (35%) and shrubs (33%) constituted the greatest proportions, followed by herbs (22%), and the remaining growth forms had a small proportion of less than 10% (Figure 3). The finding is consistent with an ethnobotanical study conducted in Uganda (Omara et al. 2020), which reported that trees and shrubs were the most dominant plant growth forms used for making herbal remedies against snakebites. The predominance of trees and shrubs in treating snakebites could be due to their accessibility throughout the year, local socio-cultural beliefs, and the practice of healers in treating snakebites (Asmeron et al. 2021; Kacholi and Amir 2022). Also, the frequent use of the two growth forms indicates that the locals are conversant with using higher plants in the formulation and preparations of herbal remedies (Kacholi and Amir 2022).

### Plant parts used

**Figure 4.**
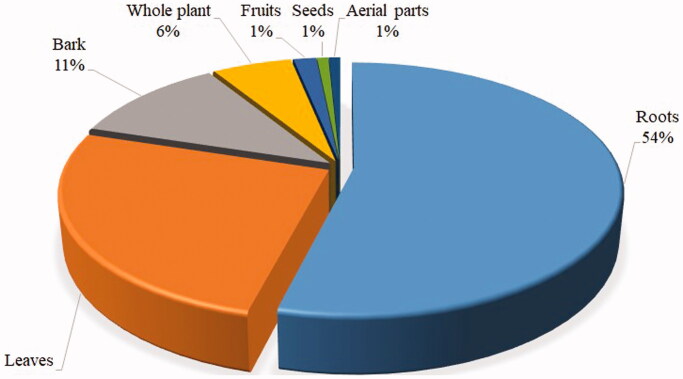
Plants parts used for preparations of herbal remedies against snakebites.

This review observed that locals in Tanzania use different plant parts to treat snakebites. Roots were the most frequently used plant part (54%), followed by leaves (26%), bark (11%) and whole plant (6%). Other parts, such as fruits, seeds and aerial parts, were rarely used (Figure 4). The common use of roots for treating snakebites was also reported in Kenya (Omara 2020), Ethiopia (Yirgu and Chippaux 2019), Uganda (Omara et al. 2020) and India (Upasani et al. 2017). The regular use of roots and leaves in antivenin preparations is a characteristic feature of traditional antivenin therapy (Owuor and Kisangau 2006; Yirgu and Chippaux 2019); that is why some of these medicinal plants are named ‘*snakeroot*’ in some rural communities.

The recurrent use of roots is also reported in treating various ailments in other countries apart from snakebites (Maroyi 2013; Jima and Megersa 2018; Mathibela et al. 2019; Hu et al. 2020; Kacholi and Amir 2022). Plant roots are believed to possess more bioactive compounds than other parts (Chinsembu 2016; Tugume and Nyakoojo 2019). The over exploitation of roots for herbal preparations may endanger plants’ existence, especially when uprooting (Kacholi and Mvungi 2021). Plants whose roots are preferred for medicinal purposes have been reported to be the most threatened species (Cunningham 2001). Thus, this study suggests that local and traditional healers’ awareness of harvesting and conserving medicinal plants is paramount.

### Preparation and administration of remedies

**Figure 5.**
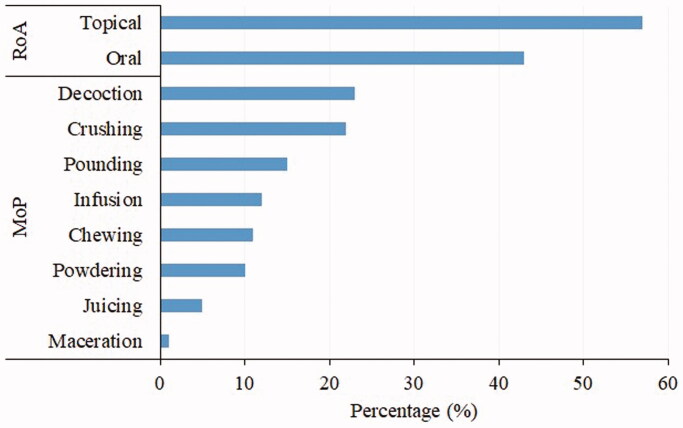
Modes of preparation (MoP) and Routes of administration (RoA) of snakebite remedies.

The treatment of snakebite in most of the areas in Tanzania involves mono-preparations of plant extracts, while in a few cases, mixtures of various plants and parts are used to prepare the antidotes. The common mode of preparation of herbal remedies is decoction (23%), followed by crushing (22%) and pounding (15%) (Figure 5). Other ethnobotanical studies (Yirgu and Chippaux 2019; Omara et al. 2020) reported decoction as a dominant preparation technique in preparing herbal remedies for snakebite management. On the other side, the most common route of administration of the medicinal plants was topical (57%), followed by oral (43%). Other previous studies showed that herbal remedies could be internal or external, whereas the intake of the plant extract mainly achieves the former, and the latter involves the application of the remedy to the bite site (Snow et al. 1994; Omara 2020). It should be noted that the study has collected limited information on how remedies are prepared and administered as 35.7% of the reported species lack the information (Table 1). Thus, it suggests that a significant effort is still needed to gather information on the mode of preparation and administration route.

### Pharmacological evidence against snake venoms

Various pharmacological studies (Núñez et al. 2004; Mali 2010; Sonibare et al. 2016) have proven the wide use of medicinal plants, which revealed that different plant metabolites could antagonize the activity of various crude venoms and purified toxins. For instance, Solanaceae is reported to possess atropine, an alkaloid which inhibits the activity of green and dark mamba (*Dendroaspis angusticeps* A. Smith (Elapidae) and *D. polylepis*) venoms by blocking cholinergic nerve terminals (Omara et al. 2020). Additionally, the family Aristolochiaceae contains aristolochic acid, an alkaloid that acts similarly to atropine (Kini 2005; Kemparaju and Girish 2006).

Moreover, the reported species in the present study have been reported elsewhere to have antivenin activities. For instance, the extract from combined roots, bark and leaves of *Securidaca longipedunculata* Fresen (Polygalaceae) (Sanusi et al. 2014), stem bark of *Commiphora africana* (A.Rich.) Engl. (Burseraceae) (Isa et al. 2015), folium extract of *Dichrostachys cinerea* (L.) Wight et Arn. (Fabaceae) (Agusi and Ogbunachi 2018), roots of *Capparis tomentosa* Lam. (Capparaceae) and *Ziziphus mucronata* Wild. (Rhamnaceae) (Molander et al. 2014), and those from the whole plant of *Euphorbia hirta* L. (Euphorbiaceae) inhibit venom activities of *N. nigricollis*. Also, extracts from leaves and roots of *A. senegalensis* inhibit venom activities of *Echis ocellatus* Stemmler (Viperidae), *N. nigricollis* and *B. arietans* (Molander et al. 2014), whereas extracts from aerial parts and roots of *Cissampelos pareira* L. var. orbiculata (DC.) Miq. (Menispermaceae) are reported to neutralize venom activity of *Bothrops diporus* Cope (Viperidae) (Verrastro et al. 2018). Leaves and roots extracts of *Cassia occidentalis* L. (Fabaceae) are reported to inhibit venom activities and accelerate wound healing caused by *Bothrops moojeni* Hoge (Viperidae) (Molander et al. 2014), while the roots and bark extract of *Acalypha fruticosa* Forssk. (Euphorbiaceae) (Molander et al. 2014), and *Paullinia pinnata* (Iful 2008; Sanusi et al. 2014) are reported to inhibit venom activities of *Echis carinatus* Schneider (Viperidae). Therefore, the present study highlights the wealthy knowledge locals in Tanzania possess in dealing with snakebites. It also suggests that further pharmacological scrutiny of the recorded medicinal plants is imperative in understanding bioactive compounds that can be used to prepare antivenin in modern science.

## Conclusions

This review presents compiled information on medicinal plant species used to treat snakebites in Tanzania. One hundred and nine medicinal plants representing 48 families were documented. Fabaceae and Euphorbiaceae were the families with the highest number of antivenom plants. Trees and shrubs were the most preferred growth forms to prepare herbal remedies for snakebites, and root was the most used plant part. Despite the diversity of plant species used to treat snakebites problems in Tanzania compiled in this review, few have been analysed for their bioactive components and potential for developing modern drugs. Therefore, effort should be geared towards this area to provide scientific information to develop snakebite drugs and solve the major health challenge. We believe that the data presented in this review will provide baseline information for future research on developing modern drugs for treating snakebite.

## Author contributions

Conceptualization and data collection, D.S.K. and N.G.M.; data analysis and manuscript writing, D.S.K., O.J.K., N.G.M. and H.A.M.; manuscript revision, D.S.K. and N.G.M.
